# Smoking, leisure-time exercise and frequency of self-reported common cold among the general population in northeastern China: a cross-sectional study

**DOI:** 10.1186/s12889-018-5203-5

**Published:** 2018-02-27

**Authors:** Ge Zhou, Hongjian Liu, Minfu He, Mengjia Yue, Ping Gong, Fangyuan Wu, Xuanxuan Li, Yingxin Pang, Xiaodi Yang, Juan Ma, Meitian Liu, Jinghua Li, Xiumin Zhang

**Affiliations:** 1 Department of Epidemiology and Biostatistics, School of Public Health, Jilin University, Changchun, China; 2 Department of Social Medicine and Health Management, School of Public Health, Jilin University, Changchun, China

**Keywords:** The common cold, Leisure-time exercise, Passive smoking, Smoking

## Abstract

**Background:**

Physical activity (PA) and smoking have been reported to be associated with the duration and severity of common cold symptoms. However, few studies have addressed the associations between the frequency of leisure-time exercise, cigarette smoking status and the frequency of the common cold in a cold area. This study was designed to investigate these issues in northeastern China.

**Methods:**

This cross-sectional study included individuals who participated in a regular health examination conducted in Jilin Province, China. Information on episodes of the common cold, the frequency of leisure-time exercise and cigarette smoking status in the past year were collected by self-administered health questionnaires. Ordinal logistic regression models were used to analyse the associations between the frequency of leisure-time exercise, cigarette smoking status and the retrospective frequency of common cold.

**Results:**

A total of 1413 employees participated in the study, with an average age of 38.92 ± 9.04 years and 44.4% of them were male. Of all participants, 80.8% reported having experienced the common cold in the past year. After adjustment, the risk of suffering from the common cold more than once (odds ratios (ORs), 1.59; 95% confidence interval (CI), 1.27–1.99) in passive smokers was 1.59 times as high as that in non-smokers. Nevertheless, the results of the adjusted analysis showed no statistically significant relation between current smoking and the frequency of the common cold. A high frequency of leisure-time exercise (≥3 days/week) was associated with a 26% reduced risk of having at least one episode of the common cold (OR, 0.74; 95% CI, 0.55–0.98) compared with a low frequency group (< 4 days/month). For current and passive smokers, the protective effect of a high frequency of leisure-time exercise appears not to be obvious (current smokers: OR, 0.68; 95% CI, 0.33–1.43; passive smokers: OR, 1.15; 95% CI, 0.69–1.93).

**Conclusion:**

Passive smoking was associated with a higher risk of having self-reported common cold at least once, while a high frequency of leisure-time exercise was related to a lower risk of reporting more than one episode of the disease in Chinese.

## Background

As one of the most prevalent infectious illnesses, the common cold is estimated to occur an average of 2–5 times for adults and 6–10 times for children each year in the general healthy population [1]. It is usually thought that the common cold, although not a severe disease, is an infection attributed to more than 200 types of viruses or bacteria [2]. Causes of the common cold are difficult to be detected because of its various clinical symptoms and the variability of microbial virulence caused by antibiotic resistance [3, 4]. Therefore, effective prevention towards reduced clinical symptoms and duration of the common cold is still limited. The common cold also has the potential to bring those suffering from respiratory diseases a certain degree of difficulty in controlling and relieving their clinical symptoms, such as worsening asthma [5, 6] and chronic obstructive pulmonary disease (COPD) [7, 8]. Furthermore, the common cold is an issue that accounts for a substantial economic burden [9] and influences normal daily activities, such as low work productivity and absenteeism due to sickness [10–12].

Smoking and exercise are modifiable lifestyle factors of maintaining health. Both smoking and passive smoking were not only related to an increased risk of various malignancies [13–15] and degenerative disorders of the cardiovascular system [16, 17] but also associated with an increased predisposition to developing the upper respiratory tract infections (URTIs) by microbial pathogens [18–24]. Although previous studies have examined the relationship between passive smoking and the incidence of the common cold in women and children [21–23], few studies have examined the association in the general population. The relationships of active smoking, severity of active smoking and frequency of common cold remain controversial and remain to be confirmed.

Physical activity (PA) was also associated with a susceptibility to URTI, but most existing studies have been performed in the elderly [25–27] and athletes [28, 29]. It has been suggested that the risk of URTI was increased for individuals engaging in extreme physical exertion [29, 30]. However, little is known about effects of moderate-intensity levels of exercise on the frequency of the common cold in predominately nonathletic population. In comparison with athletes and elders, individuals in the general individuals, especially the occupational population, are more likely to engage in moderate levels of leisure-time exercise and have less leisure-time to exercise regularly. Thus, examining the role of leisure-time exercise could urge young and middle-aged individuals to exercise, to some extent.

Northeastern of China is thought to be at high risk of respiratory disease, mainly due to exposure to the cold air in the long winter season [31], air pollutants caused by winter coal-fired heating [32–34] and inadequate vitamin D, which can affect immune function due to fewer outdoor activities in winter [35]. Therefore, the main objective of this study was to examine the influences of active or passive cigarette smoking and the frequency of leisure-time exercise on the retrospective frequency of the common cold in a cold area in northeastern China. Furthermore, we further explore the potential interaction between leisure-time exercise and cigarette smoking status on the common cold.

## Methods

### Participants

This cross-sectional study was conducted at the medical examination centre of the First Hospital of Jilin University, northeastern China, from June to September in 2016. All staff members of banks participating in regular physical examinations were invited to participate during the study period. The study followed a common set of procedures, which included physical examinations, blood and urine screenings and self-administered health questionnaires. A total of 1530 employees accepted the invitation to participated in the questionnaire survey. Of these 1530 employees, the data of 69 employees were excluded because of missing data, and that of 48 retirees were also excluded, which resulted in a final sample size of 1413 for the present analyses. The data were collected from regular medical examination reports and self-administered questionnaires in these employees. All participants were given informed consent prior to being enrolled in the study. Characteristics of the participants are reported in Tables 1 and 2.

**Table 1:** Sample basic characteristics according to the categorized cigarette smoking status

Variables	Sample (*n* = 1413)	Cigarette smoking categories				
		Non-smokers (*n* = 641)	Passive smokers (*n* = 476)	Past smokers (*n* = 82)	Light current smokers (*n* = 113)	Heavy current smokers (*n* = 101)
Gender, n (%)**						
Male	627(44.4)	185(28.9)	155(32.6)	78(95.1)^△^	108(95.6)^△^	101(100.0)^△^
Age, y**	38.92 ± 9.04	38.07 ± 8.83	38.63 ± 9.32	43.95 ± 8.0^△^	38.81 ± 8.81	41.75 ± 8.36^△^
Ethnicity, n (%)						
Han	1293(91.5)	591(92.2)	426(89.5)	75(91.5)	107(94.7)	94(93.1)
Education levels, n (%)*						
Senior school or less	83(5.9)	33(5.1)	23(4.8)	11(13.4)	8(7.1)	8(7.9)
Junior college	326(23.1)	137(21.4)	113(23.7)	23(28.0)	24(21.2)	29(28.7)
Bachelor degree	799(56.5)	365(56.9)	270(56.7)	42(51.2)	64(56.6)	58(57.4)
Master degree or greater	205(14.5)	106(16.5)	70(14.7)	6(7.3)^△^	17(15.0)	6(5.9)
BMI**	23.90 ± 5.36	23.70 ± 6.89	23.20 ± 3.52	25.90 ± 3.2^△^	25.60 ± 3.21^△^	25.40 ± 3.26^△^
Frequency of drinking (days/week), n (%)**						
0	859(60.8)	481(75.0)	293(61.6)	26(31.7)	24(21.2)	35(34.7)
1–2	468(33.1)	143(22.3)	165(34.7)	47(57.3)	69(61.1)	44(43.6)
≥ 3	86(6.1)	17(2.7)	18(3.8)^△^	9(11.0)^△^	20(17.7)^△^	22(21.8)^△^
Sleep duration, n (%)**						
< 7 hours	402(28.5)	168(26.2)	130(27.3)	22(26.8)	52(46.0)	30(29.7)
7–8 hours	561(39.7)	243(37.9)	195(41.0)	37(45.1)	38(33.6)	48(47.5)
≥ 8 hours	450(31.8)	230(35.9)	151(31.7)	23(28.0)	23(20.4)^△^	23(22.8)
SRSS, n (%)						
≥ 23	594(42.0)	257(40.1)	215(45.2)	25(30.5)	52(46.0)	45(44.6)
Self-rated health, n (%)						
Poor	168(11.9)	67(10.5)	61(12.8)	7(8.5)	15(13.3)	18(17.8)
Fair	718(50.8)	317(49.5)	244(51.3)	45(54.9)	66(58.4)	46(45.5)
Good	527(37.3)	257(40.1)	171(35.9)	30(36.6)	32(28.3)	37(36.6)
Self-perceived life stress, n (%)**						
Low	217(15.4)	108(16.8)	69(14.5)	14(17.1)	12(10.6)	14(13.9)
Fair	751(53.1)	370(57.7)	243(51.1)	43(52.4)	49(43.4)	46(45.5)
High	445(31.5)	163(25.4)	164(34.5)^△^	25(30.5)	52(46.0)^△^	41(40.6)^△^
Self-perceived work stress, n (%)*						
Low	165(11.7)	76(11.9)	56(11.8)	12(14.6)	12(10.6)	9(8.9)
Fair	615(43.5)	306(47.7)	183(38.4)	40(48.8)	42(37.2)	44(43.6)
High	633(44.8)	259(40.4)	237(49.8)^△^	30(36.6)	59(52.2)	48(47.5)
SAS, n (%)*						
≥ 50	219(15.5)	87(13.6)	89(18.7)	7(8.5)	15(13.3)	21(20.8)
SDS, n (%)*						
≥ 50	561(39.7)	236(36.8)	200(42.0)	28(34.1)	45(39.8)	52(51.5)^△^
NE**	3.78(3.10, 4.56)	3.70(3.07, 4.49)	3.73(3.00, 4.47)	3.56(3.09, 4.33)	3.94(3.22, 4.69)	4.78(3.84, 5.76)^△^
MO**	0.29(0.21, 0.39)	0.28(0.21, 0.37)	0.29(0.22, 0.39)	0.30(0.21, 0.39)	0.30(0.21, 0.42)	0.42(0.28, 0.51)^△^
LY**	2.15(1.83, 2.54)	2.13(1.81, 2.52)	2.10(1.77, 2.43)	2.14(1.80, 2.50)	2.25(1.97, 2.77)^△^	2.47(2.10, 2.87)^△^

Values are shown as the mean ± SD or median (interquartile range) for continuous data and number and proportions for categorical data

*P*-values were calculated by analysis of variance or Kruskal-Wallis *H* tests for continuous variables and *χ*^*2*^-tests for categorical variables

Superscript numbers denote statistically significant post hoc tests: ^△^≤ 0.05/4 for the difference between current, past, passive smokers and non-smokers. Current, past and passive smokers were compared with non-smokers

*Statistical significance: *P* < 0.05

**Statistical significance: *P* < 0.001

**Table 2:** Sample basic characteristics according to the categorized frequency of leisure-time exercise

Variables	Sample (n = 1413)	Frequency of leisure-time exercise		
		Low (< 4 days/month) (*N* = 375)	Medium (1–2 days/week) (*N* = 624)	High (≥3 days/week) (*N* = 414)
Gender, n (%)**				
Male	627(44.4)	141(34.1)	285(45.7)	201(53.6)
Age, y**	38.92 ± 9.04	35.25 ± 7.55	38.44 ± 8.73	43.79 ± 8.94
Ethnicity, n (%)*				
Han	1293(91.5)	370(89.4)	571(91.5)	352(93.9)
Education levels, n (%)**				
Senior school or less	83(5.9)	10(2.4)	36(5.8)	37(9.9)
Junior college	326(23.1)	80(19.3)	147(23.6)	99(26.4)
Bachelor degree	799(56.5)	268(64.7)	336(53.8)	195(52.0)
Master degree or greater	205(14.5)	56(13.5)	105(16.8)	44(11.7)
BMI**	23.90 ± 5.36	23.10 ± 3.95	24.30 ± 6.92	24.40 ± 3.18
Drinking categories (days/week), n (%)*				
0	859(60.8)	270(65.2)	365(58.5)	224(59.7)
1–2	468(33.1)	125(30.2)	223(35.7)	120(32.0)
≥ 3	86(6.1)	19(4.6)	36(5.8)	31(8.3)
Sleep duration, n (%)*				
< 7 hours	402(28.5)	115(27.8)	168(26.9)	119(31.7)
7–8 hours	561(39.7)	153(37.0)	256(41.0)	152(40.5)
≥ 8 hours	450(31.8)	146(35.3)	200(32.1)	104(27.7)
SRSS, n (%)*				
≥ 23	594(42)	189(45.7)	260(41.7)	145(38.7)
Self-rated health, n (%)				
Poor	168(11.9)	82(19.8)	59(9.5)	27(7.2)
Fair	718(50.8)	216(52.2)	353(56.6)	149(39.7)
Good	527(37.3)	116(28.0)	212(34.0)	199(53.1)
Self-perceived life stress, n (%)*				
Low	217(15.4)	52(12.6)	82(13.1)	83(22.1)
Fair	751(53.1)	217(52.4)	356(57.1)	178(47.5)
High	445(31.5)	145(35.0)	186(29.8)	114(30.4)
Self-perceived work stress, n (%)**				
Low	165(11.7)	30(7.2)	56(9.0)	79(21.1)
Fair	615(43.5)	171(41.3)	292(46.8)	152(40.5)
High	633(44.8)	213(51.4)	276(44.2)	144(38.4)
SAS, n (%)**				
≥ 50	219(15.5)	92(22.2)	87(13.9)	40(10.7)
SDS, n (%)*				
≥ 50	561(39.7)	188(45.4)	238(38.1)	135(36.0)
NE*	3.78(3.10, 4.56)	3.83(3.21, 4.72)	3.82(3.10, 4.57)	3.64(2.97, 4.36)
MO	0.29(0.21, 0.39)	0.29(0.21, 0.38)	0.30(0.21, 0.40)	0.29(0.21, 0.40)
LY	2.15(1.83, 2.54)	2.16(1.81, 2.52)	2.15(1.84, 2.56)	2.12(1.81, 2.51)

Values are shown as the mean ± SD or median (interquartile range) for continuous data and number and proportions for categorical data

*P*-values were calculated by analysis of variance or Kruskal-Wallis *H* tests for continuous variables and trend *χ*^*2*^-tests for categorical variables

*Statistical significance: *P* < 0.05

**Statistical significance: *P* < 0.001

### Common cold

The common cold was assessed with a single item: “How many times were you infected with the common cold in the previous year?” Response options included none, one, two, and three or more times, similar to prior studies [21, 36, 37]. We told participants about the definition and general clinical symptoms of the common cold [2] to help participants distinguish the common cold from flu-like illness and allergies before the health questionnaire survey. The frequency of the common cold was divided into four categories (none, one, two, three or more times) for analysis.

### Health-related behaviours

On the health questionnaires, participants were asked to choose their smoking status in the past year from the following options: non-active smoker, past smoker and current smoker. In addition, current smokers were required to answer an open-ended item: “How many cigarettes did you smoke per day in the past year?” Participants were also required to answer whether they were passively exposed to tobacco smoke by inhaling smog for more than 15 min at least once a week. In our study, we categorized current smokers as light smokers (< 15 cigarettes/day) and heavy smokers (≥15 cigarettes/day). Past smokers were considered to be those who quit more than one year ago, while those who quit less than one year ago were regarded as current smokers. Passive smokers were defined as those who had never smoked but were exposed to tobacco smoke. Current, past and passive smokers were compared with non-smokers who were non-active smokers without exposure to passive smoking. The status of cigarette smoking and the number of cigarettes were self-reported by the participants investigated in the study.

Leisure-time exercise was assessed according to an item designed by a reference to the National Health Interview Survey (NHIS) [38]: “Outside of work or daily activities, how often have you exercised per week in the past year that at least moderately increased your breathing and heart rate for at least 20 minutes?” The frequency of leisure-time exercise was divided into three categories: high (≥3 days/week), medium (1–2 days/week) and low (< 4 days/month). Intensity and patterns of leisure-time exercise were not recorded.

In the survey, participants were also required to report on drinking alcohol and engaging in alcohol abstinence, as well as on their frequency of drinking. With regard to the frequency of alcohol consumption, these participants chose one of the following four options: 0 days/week, 1–2 days/week, 3–5 days/week and 6–7 days/week. We combined data on the frequency of alcohol intake into three categories (0 days/week, 1–2 days/week and ≥3 days/week) for analysis. Average sleep duration at night was assessed by asking participants how many hours they slept on average at night. Sleep duration was categorized into three groups: < 7, 7–8 and ≥8 h. Sleep quality was measured by a continuous score based on 10 five-point items on the Self-Rating Scale of Sleep (SRSS). A score of more than 23 points indicates poor sleep according to the standard in SRSS (Cornbash’s alpha = 0.64) [39]. Previous study have confirmed the reliability and validity of SRSS for evaluating sleep quality in the Chinese population [40].

### Psychological measures

The Chinese versions of the Self-Rating Depression Scale (SDS) [41] and the Self-Rating Anxiety Scale (SAS) [42], prepared by Zung in 1965 and 1971, respectively, were used to measure depressive and anxious symptoms. Both measures comprised 20 self-reported items, and each item was scored on a 4-point scale. Increasing scores suggested an increasing severity of anxiety or depression symptoms. The criterion of having depressive or anxious symptoms was determined as a standardized score of 50 or more out of 80 points. Previous studies have confirmed the reliability and validity of the SDS and the SAS (Cronbach’s alpha = 0.85) for used in the Chinese population [43, 44].

Self-rated health (SRH) was measured by asking participants what they think of their health status in the previous year, with response options of very poor, poor, fair, good and very good. In addition, life and work stresses were evaluated with two questions as follows: 1) How do you feel about your work stress in the previous year? 2) How do you feel about your life stress in the previous year? Response options for the two questions included very low, low, fair, high and very high. Due to small numbers in several categories, we combined data on groups of SRH, self-rated life stress and self-rated work stress into three categories, respectively (SRH: poor, fair and good; life or work stress: low, fair and high).

### Biomarkers

Biomarkers (lymphocyte (LY), neutrophil (NE) and monocyte (MO)) of the participants were collected from regular medical examination reports.

### Standard control variables

Information on gender (female/male), age (continuous), ethnicity (dichotomized as Han/other), education levels, weight and height were collected using health questionnaires and health examinations. Educational levels were categorized into senior high school or less, junior college, bachelor’s degree and master’s degree or greater. Body mass index (BMI) was calculated as weight/height^2^ (kg/m^2^).

## Statistical analysis

The data were presented as means ± standard deviations (SD), medians (interquartile ranges) or number (proportions). Trend *χ*^*2*^-tests or *χ*^*2*^-tests for categorical variables and analyses of variance (ANOVA) or Kruskal-Wallis *H* tests for continuous variables were used to assess the differences in basic characteristics according to the categorized cigarette smoking status and frequency of leisure-time exercise. Post hoc analysis was conducted by the Bonferroni correction. Because the dependent variable of the frequency of the common cold had been classified into four categories according to their order of magnitude, ordinal logistic regression models were used to analyse the associations between cigarette smoking status, the frequency of leisure-time exercise and self-reported frequency of the common cold in the previous year. The odds ratios (ORs) and 95% confidence intervals (CI) were adjusted with the following potential confounding factors: gender, age, ethnicity, education levels, BMI, drinking status, sleep duration, SRSS, SRH, self-perceived work stress, self-perceived life stress, SAS, SDS, LY, NE and MO. The rationale for including these covariates in the model is that they could potentially affect the relationship of main variables (cigarette smoking status and the frequency of leisure-time exercise) and the common cold. ORs and 95%CI were calculated as exp.^(b)^. The criterion for statistical significance was set at *P* < 0.05. The data were analysed using SPSS Statistics for Windows, version 22.0 (IBM Corp, Armonk, New York, USA).

## Results

### Basic characteristics

In this study, male employees accounted for 44.4% of the sample. Of all participants, 80.8% reported catching a common cold in the past year, and 37.2%, 31.6% and 12.1% experienced the common cold during the previous year once, twice, and three or more times, respectively. The prevalence of the common cold was almost similar between genders (79.7% for males and 81.7% for females, *P* = 0.359). Similarly, there were no statistically significant gender differences in the frequency of the common cold (*P* = 0.274).

Table 1 shows the basic characteristics (standard controls variables, health-related behaviours, psychological measures and biomarkers) for the participants according to the categorized cigarettes smoking status. The results of the study indicate that almost all current smokers were males (97.7%), and the number of passive smokers in women was almost twice as high as that in men. Past, heavy current smokers (≥15cigarettes/day) were older than non-smokers. Current smokers reported a higher proportion of high-frequency daily alcohol intake (≥3 days/week) and high life stress than non-smokers, and a relative increase in having depressive symptoms (SDS ≥ 50) appeared only for the heavy/non-smokers. They had higher values for BMI and LY. Additionally, higher prevalences of high-frequency daily alcohol intake, high work stress and high life stress were found in passive smokers than in non-smokers.

Table 2 presents characteristics of the sample across the low, medium and high leisure-time exercise groups. Groups differed on the standard control variables. BMI, age, the proportion of males, and the number of participants of Han ethnicity, Junior college or lower education level and high frequency of daily alcohol intake (≥3 days/week) increased across low to high groups. In contrast, there were reductions in the percentages of participants having psychological problems (having high life or work stress and having anxious (SAS ≥ 50) or depressive (SDS ≥ 50) symptoms), reporting poor quality and sleeping more than 8 h with an increase in the frequency of leisure-time exercise. Higher-frequency exercisers reported a lower value of NE.

### Cigarettes smoking status and the common cold

**Fig. 1:**
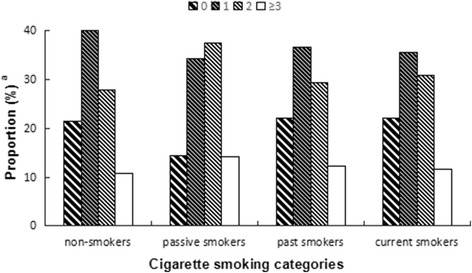
Observed association between frequency of common cold and cigarette smoking status. ^a^Represent the proportion of self-reported common cold in the categorized cigarette smoking

The proportions of participants who had the common cold more than once by cigarette smoking status (non-smokers, passive smokers, past smokers, light current smokers, heavy current smokers) were 78.5%, 85.7%, 78.0%, 78.8%, and 77.2%, respectively (data not shown). Table 3 and Fig. 1 show that the percentages of having more than two episodes of the common cold in passive, past and current smokers were higher than that in non-smokers, while the relationship between past or current smoking and the frequency of the common cold was not significant.

**Table 3:** Smoking status and frequency of leisure-time exercise according to the categorized frequency of common cold

Variables	Sample (*n* = 1413)	Frequency of common cold^a^				*P-value*
		0 (*n* = 271)	1 (*n* = 525)	2 (*n* = 446)	≥3 (*n* = 171)	
Cigarette smoking categories						
Non-smokers	641	138(21.5)	256(39.9)	178(27.8)	69(10.8)	–
Passive smokers	476	68(14.3)	163(34.2)	178(37.4)	67(14.1)	< 0.001
Past smokers	82	18(22.0)	30(36.6)	24(29.3)	10(12.2)	0.715
Light current smokers	113	24(21.2)	42(37.2)	37(32.7)	10(8.8)	0.790
Heavy current smokers	101	23(22.8)	34(33.7)	29(28.7)	15(14.9)	0.443
Frequency of leisure-time exercise						
Low < 4 days/month	414	63(15.2)	155(37.4)	132(31.9)	64(15.5)	–
Medium 1–2 days/week	624	110(17.6)	233(37.3)	205(32.9)	76(12.2)	0.206
High ≥3 days/week	375	98(26.1)	137(36.5)	109(29.1)	31(8.3)	< 0.001

^a^Episodes of self-reported common cold in the previous year

Values are shown as numbers and proportions for categorical data

*P*-values were calculated by ordinal logistic regression

Statistical significance: *P* < 0.05

Table 4 presents ORs and their 95% CI from ordinal logistic regression analyses, with the frequency of the common cold as the dependent variable. After adjustment for gender and age, the likelihood of suffering from at least one episode of the common cold was higher in passive smokers than in non-smokers (Table 4, Model 1: OR, 1.64; 95%CI, 1.32–2.03; *P* < 0.001). To determine whether other potential factors accounted for the association, we entered SRSS, SRH, health-related behaviours, psychological variables and biomarkers into ordinal logistic regression model, including cigarette smoking status and standard control variables. We observed that passive smokers had 1.59 times odds of experiencing the common cold more than once compared with non-smokers (Table 4, Model 2: OR, 1.59; 95%CI, 1.27–1.99; *P* < 0.001), and the addition of these variables did not appreciably influence the association. Nevertheless, the relationship between past or current smoking and the disease appears to be not obvious (Model 2: past smokers: OR, 1.32; 95%CI, 0.84–2.09; *P* = 0.229; light current smokers: OR, 1.05; 95%CI, 0.70–1.59; *P =* 0.801; heavy current smokers: OR, 1.26; 95%CI, 0.81–1.96; *P* = 0.295).

**Table 4:** Results of ordinal logistic regression analyses for the related factors of the common cold^a^

Variables	Statistical ordinal logistic parameters					
	Model 1^b^			Model 2^c^		
	*β*	OR(95% CI)	*P-value*	*β*	OR(95% CI)	*P-value*
Cigarette smoking categories						
Non-smokers		Ref			Ref	
Passive smokers	0.49	1.64(1.32–2.03)	< 0.001	0.46	1.59(1.27–1.99)	< 0.001
Past smokers	0.29	1.34(0.85–2.10)	0.207	0.28	1.32(0.84–2.09)	0.229
Light current smokers	0.18	1.19(0.80–1.77)	0.384	0.05	1.05(0.70–1.59)	0.801
Heavy current smokers	0.33	1.39(0.92–2.11)	0.121	0.24	1.26(0.81–1.96)	0.295
Frequency of leisure-time exercise						
Low (< 4 days/month)		Ref			Ref	
Medium (1–2 days/week)	− 0.11	0.89(0.71–1.13)	0.344	− 0.04	0.96(0.76–1.22)	0.764
High (≥3 days/week)	− 0.45	0.64(0.48–0.84)	0.001	−0.31	0.74(0.55–0.98)	0.035

^a^Episodes of self-reported common cold in the previous year

Ordinal logistic regression models were used to analyse the association of cigarette smoking status, frequency of leisure-time exercise and self-reported frequency of common cold

^b^Adjusted models control for gender and age

^c^Adjusted models control for gender, age, ethnicity, education levels, BMI, frequency of drinking, sleep duration, SRSS, SRH, self-perceived life stress, self-perceived work stress, SAS, SDS, LY, NE and MO

Statistical significance: *P* < 0.05

### Physical activity and the common cold

**Fig. 2:**
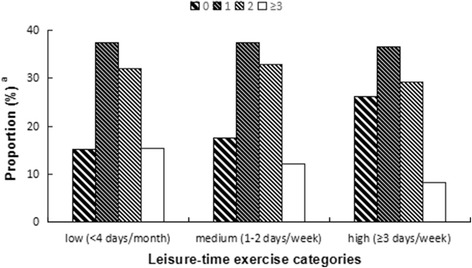
Observed association between frequency of common cold and frequency of leisure-time exercise. ^a^Represent the proportion of self-reported common cold in the categorized leisure-time exercise

When retrospective episodes of the common cold were divided by leisure-time exercise groups, at least one episode of the common cold was reported by 84.8% of the low group (< 3 days/month), 82.4% of the medium group (1–2 days/week), and 73.9% of the high group (≥3 days/week) (data not shown). Table 3 and Fig. 2 show the trend of an increase in the percentage of people who had not caught a cold in the previous year with increasing frequency of leisure-time exercise, while there was an inverse trend for the proportion of individuals experiencing three or more episodes of the common cold.

Inspection of the ORs adjusted for gender and age reveals a significant association between the risk of the common cold and the frequency of leisure-time exercise. The risk of experiencing more than one episode of the common cold during a 12-month prospective period was 36% lower in the high vs. low exercise groups (Table 4, Model 1 high group: OR, 0.64; 95%CI, 0.48–0.84; *P* = 0.001). After multivariable adjustment, the high group of leisure-time exercise was associated with a 26% reduced risk of having at least one episode of the common cold in comparison to the low group. Inclusion of these variables resulted in a 10.0% increment in the OR of leisure-time exercise associated with self-reported common cold in the high vs. low groups for leisure-time exercise levels, but a relevant effect size remained robust (Model 2 high group: OR, 0.74; 95%CI, 0.55–0.98; *P* = 0.035).

### The effect of the interaction between leisure-time exercise and cigarette smoking on the common cold

Table 5 presents the risk of the common cold for leisure-time exercise by cigarette smoking status. These results indicate that an inverse association between the high group of leisure-time exercise and the risk of experiencing at least one episode of the common cold was significant in non-smokers (OR, 0.54; 95%CI, 0.35–0.84; *P* = 0.006), especially in women (OR, 0.48; 95%CI, 0.28–0.80; *P* = 0.005). For past smokers, the protective effect of a high frequency of leisure-time exercise appears to approach marginal significance (OR, 0.22; 95%CI, 0.05–0.96; *P* = 0.045), while the frequency of leisure-time exercise was found to have no statistically significant influence on the risk of the common cold in current and passive smokers. (current smokers: OR, 0.68; 95%CI, 0.33–1.43; *P* = 0.313; passive smokers: OR, 1.15; 95%CI, 0.69–1.93; *P* = 0.595).

**Table 5:** Leisure-time exercise and OR of common cold by cigarette smoking categories

Frequency of leisure-time exercise	Non-smokers			Passive smokers			Past smokers			Current smokers		
	*β*	OR(95% CI)^a^	*P-value*	*β*	OR(95%CI)^a^	*P-value*	*β*	OR(95% CI)^a^	*P-value*	*β*	OR(95% CI)^a^	*P-value*
All sample												
Low (< 4 days/month)		Ref			Ref			Ref			Ref	
Medium (1–2 days/week)	−0.204	0.82(0.58–1.15)	0.246	0.079	1.08(0.71–1.65)	0.713	0.917	2.50(0.60–10.46)	0.209	−0.227	0.80(0.41–1.53)	0.495
High (≥3 days/week)	−0.615	0.54(0.35–0.84)	0.006	0.140	1.15(0.69–1.93)	0.595	−1.529	0.22(0.05–0.96)	0.045	−0.381	0.68(0.33–1.43)	0.313
Men												
Low (< 4 days/month)		Ref			Ref			Ref			Ref	
Medium (1–2 days/week)	−0.003	1.00(0.49–2.02)	0.994	−0.724	0.48(0.20–1.20)	0.116	0.814	2.26(0.53–9.71)	0.274	−0.217	0.80(0.41–1.57)	0.523
High (≥3 days/week)	−0.128	0.88(0.37–2.09)	0.772	−0.200	0.82(0.31–2.17)	0.688	−1.532	0.22(0.05–0.96)	0.045	−0.391	0.68(0.32–1.43)	0.306
Woman												
Low (< 4 days/month)		Ref			Ref							
Medium (1–2 days/week)	−0.221	0.80(0.54–1.20)	0.283	0.231	1.26(0.77–2.05)	0.351		–			–	
High (≥3 days/week)	−0.744	0.48(0.28–0.80)	0.005	0.047	1.05(0.55–1.99)	0.887		–			–	

Ordinal logistic regression models were used to analyse the association of leisure-time exercise and OR of the common cold, both globally and stratified by gender

^a^Adjusted models control for gender, age, ethnicity, education levels, BMI, frequency of drinking, sleep duration, SRSS, SRH, self-perceived life stress, self-perceived work stress, SAS, SDS, LY, NE and MO

Statistical significance: *P* < 0.05

## Discussion

Poor sleep, poor self-rated health, mental stress, anxious symptoms and depressive symptoms are risk factors of URTI, as confirmed in other studies [27, 45–47]. However, compared with these factors, lifestyle strategies for URTI prevention can be relatively easy to implement and have received consistent scientific supports. We combined data from the cross-sectional study revealing that passive smoking and a high frequency of leisure-time exercise (≥3 days/week) were important correlates of the retrospective frequency of the common cold in the general population. The association was independent of socio-demographic, SRSS, SRH, health-related behaviours, psychological variables and biomarkers.

We found that the probability of suffering from at least one episode of the common cold during the previous year was higher in passive smokers than in non-smokers, while the impact of active smoking on the frequency of the common cold was not observed. The results were similar to other investigations of the association between cigarette smoking and RTI [21, 48]. In the trial by Bensenor and his colleagues in 2001 [21], the risk of more frequent colds was higher for women who were non-smokers with passively exposed to cigarette smoking (RR, 1.33; 95%CI, 1.18–1.51), while current light (<25cigarettes/day) (RR, 0.94; 95%CI, 0.81–1.10) and heavy (≥25cigarettes/day) (RR, 1.17; 95%CI, 0.81–1.10) smokers had no appreciable association with the frequency of common cold relative to women who were non-smokers without exposed to passive smoke. German and his colleagues also indicated that they did not find an association between smoking and symptomatic respiratory infection in a cohort of military recruits in Greece [48]. In addition, previous studies performed in children provide some supports for an impact, such that children who were exposed to environmental tobacco smoke had higher risk for URTI than children who lived in a smoke-free environment [22, 23].

Nevertheless, the findings presented should be interpreted with caution. Although we data fail to show that there was an increase in the frequency of the common cold in active smokers, previous studies indicated that increased damages by URTI in active smokers were attributable to increased duration and severity of the URTI symptoms [19, 21] and the restricted the efficacy of anti-infectious therapy [18, 49]. Furthermore, other earlier experimental and epidemiological studies observed that active smokers had a higher incidence of URTI [24, 50].

The discrepancy shown in our study might be explained by the limitation that the proportion of active smokers was too low (15.1%), which may have led to a false negative association for current smokers. To determine whether active smoking is a risk factor for the frequency of common cold, a sufficiently large sample size is needed to be collected in our study.

Another possible explanation for the results is that passive smokers mainly absorbed side-stream tobacco smoke drifting from lit cigarettes, and there were higher concentrations of toxic gases, including ammonia and dimethylnitrosamine, in side-stream than in mainstream smoke inhaled by active smokers [18, 51]. It has been reported that fresh side-stream smoke at concentrations commonly encountered indoors is well above the 2 mg/m^3^ reference concentration, and the level can cause respiratory tract epithelium damage [52]. In addition, non-smokers were more vulnerable to effects of tobacco smoke than past and current smokers [21], because their cardiopulmonary function is probably less “adaptable to tobacco smoke”. Tobacco smoke has a suppressive effect on the protective functions of immunocyte and adaptive immune mechanisms, and had a direct effect on microbial pathogens to enhance the probability of infectious disease, specifically promoting microbial virulence and antibiotic resistance [18, 53].

Regular moderate PA enhanced resistance to URTI, which can reduce the number of episodes, clinical symptoms and duration of URTI [27, 36, 54]. A cohort study performed in healthy adults aged 20–70 years indicated that there was a “J”-shaped relationship of PA and the risk of URTI, and high levels of moderate-vigorous activity were associated with a reduced risk for URTI relative to low levels of activity [36]. An epidemiological study conducted by Nieman et al. showed that the frequency of near-daily aerobic activity was correlated with reduced severity and duration with URTI symptoms during a 12-week period [54]. By contrast, among the frail elderly nursing home population, a 32-week exercise intervention was not associated with a change in the incidence of URTI [26]. Moreover, an earlier study performed in elderly women also concluded that 12 weeks of moderate cardiorespiratory exercise did not bring about an improvement in immune function [25].

Our study supported the opinion that in the general healthy Chinese population, leisure-time moderate exercise at least three times per week was inversely related to the risk of experiencing at least one episode of the common cold, while a “J”-shaped association between leisure-time moderate and the risk of the common cold was not found. Given that the participants investigated in our study were staff members at a bank, a professional population, few individuals performed heavy physical labour or had an opportunity to participate in high levels of exercise training. Thus, the effects of high levels of exercise training on the risk of the common cold could not be found, as implied by the latter part of the “J”- shaped curve. Findings from the investigation demonstrated that the risk of having one or more episodes of the common cold was reduced by 26% in the high vs. low groups of leisure-time exercise after controlling for confounders. A four months follow-up period in the general population aged 20–60 years showed that high levels of PA (≥55 MET•h•d-1) were associated with an 18% reduced risk (IRR, 0.82; 95% CI, 0.69–0.98) of self-reported URTI compared with low level group(< 45 MET•h•d-1) [55]. A study of the symptomatology of URTI and habitual PA performed in elderly indicated that the risk for having at least three URTI episodes per year was 9% lower in subjects with higher 7-day recall moderate scores (OR, 0.91; 95% CI, 0.83–0.99) [27]. The reduction in the risk of the common cold with a high frequency of leisure-time exercise had more significance in our study is probably because the individuals we investigated are predominately nonathletic adults with sedentary lifestyle. Moderate PA has been deemed to improve immune competence in a manner, and the mechanisms for the effect of moderate exercise on the reduction of URTI risk are still being explored and debated. The physiological mechanism might be explained by the fact that immune system function is partially enhanced via increasing the activity of natural killer cells and the function of superior T cells [56]. Moreover, recent data suggest that moderate-intensity exercise can increase the secretion of immunoglobulin (Ig) A [57, 58], and salivary IgA plays a major role in the human mucosal immune system [59].

In addition, our data indicate that a high frequency of leisure-time exercise was associated with a reduced risk of experiencing at least one episode of the common cold in non-smokers, and the protective effect of a high level of leisure-time exercise appears to approach marginal significance in past smokers. However, we did not see that leisure-time exercise reduced the negative effects of cigarette smoke on common cold risk, as suggested by Hemila et al. [60]. The findings in their study showed that moderate PA had no material association with a lower common cold risk in middle-aged male smokers. A study performed in postmenopausal women compared moderate-intensity exercise individuals with those engaging in stretching sessions, finding that the incidence of URTI can be reduced in non-smoking women [61]. In contrast, the abovementioned study conducted by Fondell et al. indicated that a high level of PA was associated with a reduced risk of self-reported common cold for both smokers and non-smokers [55]. This difference is possibly explained by the fact that the association between leisure-time exercise and the common cold was modified by smoking status. Passive smokers were at a relatively high risk, irrespective of how much exercise they performed. Non-smokers and past smokers have eliminated the negative effects of cigarette smoke on the common cold. Therefore, the risk of the common cold was reduced as the frequency of moderate exercise increased. It is noteworthy that this phenomenon was not observed in current smokers. Considering that previous studies have confirmed the effect of active smoking on cold symptoms [19, 21], it is necessary to further explore the association between active smoking and the frequency of the common cold in our study.

Several shortcomings of the present study should be acknowledged. First, it was an observational study that relied on self-reports of the common cold without confirmation by laboratory examination. However, it is the subjective symptoms that affect the individual’s life and work status, and therefore the subjective outcome is more relevant for the purpose of public health [60]. Furthermore, episodes of the common cold and the exposure to main variables (leisure-time exercise and cigarettes smoking status) were also self-reported. Therefore, recall bias may exist in the process of information collection. Second, the associations among common cold risk and the intensity, duration and types of leisure-time exercise patterns, as well as the impact of daily activities, were not analysed. In fact, as mentioned before, considering the professional nature of participants investigated in our study, the likelihood of engaging in extreme intensity of leisure-time exercise would be slim. Third, we controlled for the SRH, SRSS, socio-demographic variables, psychological variables, biomarkers and other health-related behaviours, but there were still other possibilities we had not covered, for instance, the number of people with whom the participants may share an office or apartment and the health status of apartment mates and colleagues [62].

## Conclusion

In summary, our study revealed that passive smoking was associated with higher risk of having at least one episode of a self-reported common cold during the previous year. Nevertheless, active smoking was not significantly related to the frequency of the common cold. In addition, a high frequency of leisure-time exercise (≥3 days/week) in the general population of healthy adults was associated with a lower risk of experiencing the self-reported common cold more than once than a low frequency group. For smokers and passive smokers, the protective effect of a high level of leisure-time exercise on the risk of the common cold does not appear to be obvious.

## Abbreviations

BMI: Body mass index
LY: Lymphocyte
MO: Monocyte
NE: Neutrophil
PA: Physical activity
SAS: Self-Rating Anxiety Scale
SDS: Self-Rating Depression Scale
SRH: Self-rated health
SRSS: Self-Rating Scale of Sleep
